# The non-octarepeat copper binding site of the prion protein is a key regulator of prion conversion

**DOI:** 10.1038/srep15253

**Published:** 2015-10-20

**Authors:** Gabriele Giachin, Phuong Thao Mai, Thanh Hoa Tran, Giulia Salzano, Federico Benetti, Valentina Migliorati, Alessandro Arcovito, Stefano Della Longa, Giordano Mancini, Paola D’Angelo, Giuseppe Legname

**Affiliations:** 1 Department of Neuroscience, Scuola Internazionale Superiore di Studi Avanzati (SISSA), Trieste, Italy; 2 Structural Biology Group, European Synchrotron Radiation Facility (ESRF), Grenoble, France; 3 Department of Chemistry, Sapienza University of Rome, Rome, Italy; 4 Istituto di Biochimica e Biochimica Clinica, Università Cattolica del Sacro Cuore, Rome, Italy; 5 Department of Medicine, Public Health, Life and Environmental Science, University of L’Aquila, Coppito Aquila, Italy; 6 Scuola Normale Superiore, Pisa, Italy; 7 Istituto Nazionale di Fisica Nucleare (INFN) sezione di Pisa, Pisa, Italy; 8 ELETTRA - Sincrotrone Trieste S.C.p.A, Trieste, Italy

## Abstract

The conversion of the prion protein (PrP^C^) into prions plays a key role in transmissible spongiform encephalopathies. Despite the importance for pathogenesis, the mechanism of prion formation has escaped detailed characterization due to the insoluble nature of prions. PrP^C^ interacts with copper through octarepeat and non-octarepeat binding sites. Copper coordination to the non-octarepeat region has garnered interest due to the possibility that this interaction may impact prion conversion. We used X-ray absorption spectroscopy to study copper coordination at pH 5.5 and 7.0 in human PrP^C^ constructs, either wild-type (WT) or carrying pathological mutations. We show that mutations and pH cause modifications of copper coordination in the non-octarepeat region. In the WT at pH 5.5, copper is anchored to His96 and His111, while at pH 7 it is coordinated by His111. Pathological point mutations alter the copper coordination at acidic conditions where the metal is anchored to His111. By using *in vitro* approaches, cell-based and computational techniques, we propose a model whereby PrP^C^ coordinating copper with one His in the non-octarepeat region converts to prions at acidic condition. Thus, the non-octarepeat region may act as the long-sought-after prion switch, critical for disease onset and propagation.

The pathological form of the cellular prion protein (PrP^C^) plays a central role in a variety of human and animal neurodegenerative disorders, known collectively as prion diseases or transmissible spongiform encephalopathies (TSE). These disorders arise from misfolding of the mainly α-helical folded PrP^C^ to a β-sheet enriched, protease-resistant and infectious isoform, termed PrP^Sc^ or prion. TSE uniquely manifest as sporadic, genetic and iatrogenic forms, and they include most notably Creutzfeldt-Jakob disease in humans, bovine spongiform encephalopathy in cattle, scrapie in sheep and goat, and chronic wasting diseases in cervids^1^.

Structural information from NMR spectroscopy and X-ray crystallography studies shows that the C-terminal PrP^C^ domain (residues 128 to 231, hereafter in human PrP^C^ numbering) adopts a predominantly α-helical conformation with small β-sheet content^2^,^3^ (Fig. 1a). The N-terminal half of the protein (residues 23 to 127) does not adopt any identifiable folding structure in solution^4^. The latter features four histidine (His) residues within the highly conserved octapeptide repeats (OR) (residues 60–91). This region is able to coordinate up to four copper ions with distinct coordination geometries and high affinity^5^. Two additional His residues able to bind copper are found in the 92–111 region, including a charged segment (residues 92–111) denoted as non-OR region^6^ adjacent to a hydrophobic segment (residues 112–127) thought of playing a role during prion conversion^7^. The amino acidic sequence spanning residues 90 to 127 shows high degree of conservation among different mammalian species, with local amino acidic substitutions clustered in the non-OR region (Fig. 1b).

Unlike PrP^C^, the PrP^Sc^ structure features a significant –if not all- content of β-sheet secondary structure motives^8^. These unique structural features of PrP^Sc^ are responsible for its physicochemical properties, including insolubility in non-ionic detergents, protease-K (PK) resistance and aggregation propensity^1^. The insoluble and heterogeneous nature of PrP^Sc^ renders its structural characterization extremely difficult, and to date several prion structural models have been proposed^9^.

Yet, normal PrP^C^ function(s), as well as the structural and molecular mechanisms leading to PrP^Sc^ conversion and transmission, remain largely unknown^10^. PrP^C^ is tethered to the extracellular cell surface *via* a modified lipid anchor linked to the C-terminus, and it displays highest expression levels at the presynaptic membrane of neurons^11^. Mounting evidence suggests a central role for PrP^C^ in neuroprotection. Proposed PrP^C^ functions range from neuronal growth and differentiation^12^, synaptic plasticity^13^, cell signaling^14^, to N-methyl-D-aspartate (NMDA) receptors modulation^15^,^16^,^17^ and brain metal homeostasis^18^. The latter two functions are attributed to the PrP^C^ ability to bind copper and -to a lesser extent- other divalent cations such as zinc and manganese^19^,^20^. At the synapse, copper is released during depolarization *via* synaptic vesicles, reaching transient concentrations up to 250 μM in the synaptic cleft, thus suggesting that copper release may be an integral component of neurotransmission^21^. Copper binding to PrP^C^ promotes its endocytosis *via* clathrin-dependent pathway, arguing that this endocytic mechanism may serve to reuptake copper within the presynaptic terminal after depolarization^22^. More recent results confirmed that PrP^C^ and copper cooperatively exert neuroprotection limiting intracellular calcium accumulation through a copper-mediated S-nitrosylation of the NMDA receptor^15^.

Although copper binding seems to be involved in many physiological PrP^C^ processes, the interplay between copper and PrP^Sc^ conversion poses intriguing issues. While the OR region does not appear to be essential for prion infectivity^23^, the non-OR copper-binding site may be relevant because of its location adjacent to a region –the palindromic motif sequence AGAAAAGA- involved in structural changes during the early stage of prion conversion^24^. This regional proximity raised the question whether copper bound to the non-OR site may have an impact on prion conversion. Indeed, the interaction of copper with a peptide including both the non-OR and the palindromic motif has been shown to induce β-sheet formation and aggregation of this segment^25^,^26^. Further *in vivo* findings have supported the idea that the region encompassing residues 90–125 is involved in prion generation. In addition, this region seems to have a crucial role in preserving physiological PrP^C^ function(s). While the deletion of the OR in transgenic (Tg) mice is not toxic, the ablation of segments including the non-OR and the hydrophobic region results in diseased mouse phenotypes with cerebral disorders^27^,^28^. Interestingly, naturally occurring point mutations clustered in the non-OR region and in the palindromic motif are responsible for Gerstmann–Sträussler–Scheinker (GSS) syndrome, characterized by PrP^Sc^ amyloidal plaque deposits in the brain (Fig. 1b). These observations corroborate the idea of the non-OR as pivotal partner in both PrP^C^ function and conversion to prion.

Here, by means of extended X-ray absorption fine structure (EXAFS) spectroscopy, cell-biology approaches and molecular dynamic (MD) simulations, we have investigated how copper coordination in the non-OR region may influence prion conversion. EXAFS spectroscopy is a powerful technique for obtaining structural and chemical information about metal binding to a protein of interest, providing accurate bond length measurements within 5 Å or less. Several groups have analyzed by EXAFS copper coordination bound to recombinant PrP in the OR^29^ and non-OR regions^30^,^31^. In a previous EXAFS study we found that at pH 5.5 Cu(II) and Cu(I) bound to wild-type (WT) are specifically coordinated by the N*δ*-atom in the imidazole ring of two His residues (H96 and H111), by deprotonated amide nitrogens from adjacent residues (*e.g.* Q98), as well as by sulfur-mediated bounds from either methionine (Met) 109 or M111^30^. This coordination geometry is completely altered in the HuPrP(90–231) carrying the Q212P substitution, a mutation linked to GSS syndrome, suggesting that this mutant may have structural consequences both at the C-terminal globular domain^32^ and at the non-OR region. In order to gain insights into the role of mutations on the non-OR region, we investigated the copper coordination in the WT HuPrP(90–231) and in different mutants at both pH 7.0 and pH 5.5 – the latter mimicking the acidic endosomal compartments^33^. As model systems for the mutants we used Q212P, P102L –the prototypical GSS mutation^34^ - and H96Y –an artificial mutation devoid of one His residue involved in copper binding. EXAFS data clearly highlighted modifications of the non-OR copper-binding site induced by these mutations. To understand the physiological implications of our EXAFS data, we performed *in vitro* and cell-based approaches. We used neuronal cell models expressing 3F4-tagged murine (Mo) PrP^C^ (MoPrP) (Fig. 1b) in which the N-terminal His residues within the OR and non-OR regions were substituted by tyrosine (Tyr) and we evaluated the effect of single His to Tyr substitutions in the context of prion conversion. Intriguingly, only H96Y mutation largely promoted prion conversion, PrP accumulation in the endosomal compartments, and generation of *bona fide* infectious prion material (*e.g.* displaying partial PK-resistance and the ability to perform seeded aggregation *in vitro* and in cell). Finally, MD simulations were used to hypothesize possible structural consequences on the HuPrP structure caused by an altered copper coordination in the non-OR region. In our simulations we predicted structural facets of WT HuPrP(90–231) coordinating Cu(II) *via* either H111 or both H96 and H111. Comparison of trajectories showed that removal of H96 ligand from the Cu(II) coordination results in β-sheet enrichment in the segment spanning the non-OR region and palindromic motif.

Our study highlights the importance of the non-OR region for prion conversion and proposes a model in which PrP^C^ coordinating copper with one His residue may render PrP^C^ more prone to prion conversion at acidic pH condition.

## Materials and Methods

### Plasmids construction, protein expression and purification

The H96Y, P102L and Q212P mutations were inserted in pET-11a encoding for HuPrP(90–231) as previously described^30^. The pET-11a plasmid (Novagen) encoding for the full-length MoPrP -residues 23–231, here denoted as MoPrP(23–231)- was kindly provided by Dr. J.R. Requena (University of Santiago de Compostela, Santiago de Compostela, Spain). The H96Y mutation was inserted into the full-length MoPrP DNA by mutagenesis kit (Stratagene). The open reading frame encoding for the pre-pro MoPrP from residue 1 to 254 was amplified by PCR from genomic murine DNA and cloned by restriction-free method in pcDNA3.1(-) vector (Invitrogen). The 3F4 epitope tag and the single point mutations (H61Y, H69Y, H77Y, H85Y and H96Y in human numbering single amino acid code) were inserted by mutagenesis into the pcDNA3.1::MoPrP(1–254). The WT HuPrP(90–231), HuPrP(90–231, H96Y), HuPrP(90–231, P102L), HuPrP(90–231, Q212P) and the WT MoPrP(23–231) were expressed, purified and *in vitro* refolded according to our previous protocols^30^,^32^.

### XAS spectra data collection

Samples with 1:1 Cu(II):WT and mutants HuPrP(90–231) ratio were prepared in 25 mM NaOAc pH 5.5 and 25 mM MOPS buffer pH 7.0. The Cu(I):WT and mutants HuPrP(90–231) complexes were generated reducing Cu(II) with 40 mM ascorbate as previously described^30^. X-ray absorption spectra were recorded at the European Synchrotron Radiation Facility (ESRF) on beam line BM30B FAME^35^, under ring conditions of 6.0 GeV and 180 mA. The spectra were collected at the Cu K-edge in fluorescence mode using a solid state 30-element Ge detector, with sample orientation at 45° to incident beam. The X-ray photon beam was vertically focused by a Ni−Pt mirror, and dynamically sagittally focused in the horizontal size. The monochromator was equipped with a Si(111) double crystal, in which the second crystal was elastically bent to a cylindrical cross section. The energy resolution at the Cu K-edge is 0.5 eV. The spectra were calibrated by assigning the first inflection point of the Cu foil spectrum to 8981. All the spectra were collected at 10 K. For the Cu(II) samples photo reduction was observed and thus the beam was moved to different spots of the sample at each scan. During collection, data were continuously monitored in order to insure sample homogenity across the multiple spots collected from different sample-holder’s cells. Complete data sets were collected for samples 1 to 14 (Supplementary Table 1). For each sample, 12 spectra were recorded with a 7 s/point collection statistic and averaged. The collection time was 25 min for each spectrum.

### EXAFS data analysis

EXAFS data analysis was performed using the GNXAS code, which is based on the decomposition of the EXAFS signal (defined as the oscillation with respect to the atomic background cross-section normalized to the corresponding K-edge channel cross-section) into a summation over n-body distribution functions γ^(n)^, calculated by means of the multiple-scattering (MS) theory^36^. The analysis of the EXAFS spectra was carried out starting from the coordination model previously determined^30^. In particular, Cu(II) was found to be coordinated to H96, H111, and two additional low Z ligands (oxygen or nitrogen donors) in the inner shell and to a sulphur-donating ligand, which was assigned to M109. The final fit included also one close oxygen-donating ligand that was assumed to derive from solvent, although it could also be derived from a protein ligand such as a Q98. Based on this model, theoretical EXAFS spectra were calculated to include contributions from first shell two-body signals, and three-atom configurations associated with the His rings. The model χ(k) signal has been then refined against the experimental data by using a least-squares minimization procedure in which structural and nonstructural parameters are allowed to float. The structural parameters are bond distance (R) and bond variance (σ^2^_R_) for a two-body signal, the two shorter bond distances, the intervening angle (θ), and the six covariance matrix elements for a three-body signal. During the minimization procedures, the magnitudes of the Debye–Waller terms for the imidazole rings were assumed to increase with distance, and imidazole ring atoms at similar distances from the copper ion were assigned the same value. The EXAFS spectra were analyzed trying out different possible models including either one or two His ligands and a sulfur coordinating atom. In all cases two additional nonstructural parameters were minimized, namely, E_0_ (core ionization threshold) and S_0_^2^ (many body amplitude reduction factor). The quality of the fits was determined by the goodness-of-fit parameters^37^, R_i_, and by careful inspection of the fit of the EXAFS data, Fourier trasform, and individual EXAFS γ^(n)^ signals. To establish error limits on the structural parameters, a number of selected parameters from the fit results were statistically analyzed using two-dimensional contour plots. This analysis examines correlations among fitting parameters and evaluates statistical errors in the determination of the copper coordination structure, as previously described^36^. Briefly, parameters with highest correlation dominate in the error estimate. The results of the EXAFS fits for all the systems are given in Supplementary Table 1 and the best-fit curves are shown in Supplementary Fig. 1 for Cu(II) and Cu(I) HuPrP(90–231) at pH 7.0, as an example. The EXAFS spectra were analyzed in the *k* range between 2.4–12.6 Å^−1^ for Cu(II) and 2.5–13.0 Å^−1^ for Cu(I). In all cases S_0_^2^ was found equal to 0.9, while E_0_ was found 3 and 7 eV above the first inflection point for the Cu(II) and, Cu(I) spectra, respectively.

### Neuroblastoma cell cultures

N2a and ScN2a cells were cultured in Opti-MEM (GIBCO) media supplemented with 10% fetal bovine serum (FBS) and 1% penicillin-streptomycin, and incubated at 37 °C, 5% CO_2_. Transient transfections were performed using X-treme gene DNA transfection kit (Roche Biochemicals) according to the manufacturer guidelines. Seventy-two hours post-transfection, the cells were collected for further analysis.

### Biochemical assays on PrP^Sc^ and PrP^C^

Cell lysates were harvested in cold lysis buffer (10 mM TrisHCl pH 8.0, 150 mM NaCl, 0.5% Nonidet P-40 substitute, 0.5% sodium deoxycholate), quantified by BCA protein assay kit (Pierce) and stored at −20 °C until use. For protease-K (PK) digestion assay, quantified protein lysates were treated with PK (Roche) at 37 °C. ScN2a cell lysates were digested with 20 μg/mL of PK for 1 hour, while cell lysates from N2a cells transfected with either WT or H96Y 3F4-MoPrP constructs were digested with 2 and 5 μg/mL of PK for 30 minutes. PK digestions were stopped by adding 2 mM phenylmethyl-sulphonyl fluoride. Subsequently, the samples were ultracentrifuged at 100,000 g for 1 h at 4 °C (Optima TL, Beckman Coulter, Inc.). The pellets were resuspended in sample buffer. The glycan modifications on PrP^C^ were assessed using Endo-H and PNGase-F enzymes (New England Biolabs) according to the manufacturer instructions. Samples were loaded onto a 10% SDS-PAGE gel and immunoblotted on Immobilion PVDF (Millipore) membranes. Membranes were blocked with 5% (w/v) non-fat milk protein in TBS-T (0.05% Tween), incubated with 1:1000 anti-PrP antibody 3F4 (Covance), and developed by enhanced chemiluminescence (GE Healthcare). Band intensity was acquired using the UVI Soft software (UVITEC, Cambridge). Total PrP^C^ expression levels in N2a and ScN2a cell lysates were normalized on β-actin value using 1:10,000 anti-β-actin Peroxidase (Sigma-Aldrich). The PrP^Sc^ PK-resistance levels in all the mutants were derived using as reference the PK-resistant band intensity of the ScN2a cells transfected with WT 3F4-MoPrP. To evaluate the role of copper in prion conversion, ScN2a cells were transiently transfected with pcDNA3.1::3F4-MoPrP(1–254) WT and pcDNA3.1::3F4-MoPrP(1–254) H96Y plasmids, treated for 48 hours with increasing concentration (10, 20, 30 and 40 μM) of cuprizone (CPZ, Sigma Aldrich) and immediately collected for lyses, PK digestion and immunoblot as described above.

### Fluorescence imaging

Cells were grown on poly-L-lysine-coated coverslips for 24 hours before fixation in 4% paraformaldehyde and washed with PBS prior to blocking in 1% FBS, 0.3% Triton X-100. Cells were incubated at 4 °C for 12 hours in blocking buffer with anti-PrP primary antibodies, *i.e.* 3F4 and D18 (InPro Biotechnology) monoclonal antibodies. The following day, cells were incubated for 1 hour with secondary antibodies conjugated with AlexaFluor. For PrP^C^ cell surface detection, cells were incubated at 4 °C for 15 min and probed with 3F4 antibody. Cells were permeabilized with 0.2% Triton-X and stained with AlexaFlour-488 secondary antibody. To detect Thioflavin-S (ThS)-positive aggregates, transfected and non-transfected cells were fixed with 4% PFA/4% sucrose/1% Triton X-100 in PBS. For organelle markers, we used anti-Calnexin (ER marker), anti-EEA1 (early endosome marker), anti-Tfn (recycling endosome marker), anti-M6PR (late endosomes marker) and anti-LAMP2 (lysosomes marker) purchased from Abcam. Nuclei were stained with DAPI dye (VECTOR Laboratories). Images were acquired with a DMIR2 confocal microscope equipped with Leica Confocal Software (Leica).

### Monitoring the kinetics of *in vitro* fibril formation

To monitor the formation of ThT-positive fibrils, we used 100 μg/mL of WT full-length MoPrP stored in acetate buffer (25 mM NaOAc, 6 M GdnHCl, pH 5.5) or Tris buffer (25 mM Tris-HCl, 6 M GdnHCl, pH 7.0). To induce protein polymerization in the amyloid seeding assay (ASA) we added a preformed PrP^Sc^ seed to the reaction purified from ScN2a transiently expressing either 3F4-tagged WT MoPrP or 3F4-tagged H96Y MoPrP according to our previous protocols. Data were analyzed and figures were produced using Origin 8.6 software.

### Prion formation in N2a cells

N2a cells were transiently transfected with either 3F4-tagged WT or H96Y MoPrP and regularly passaged every 7 days up to passage (P) 8. Subsequently, the protein extracts were analyzed by PK digestion to monitor the presence of PK-resistant PrP^Sc^ levels through passages (see above). Prion formation was assessed by cell seeding experiments. Phosphotungstic acid (PTA)-extracted PrP^res^ seeds -we denoted as PrP^res^ the material generated by N2a cells transfected with 3F4-tagged H96Y MoPrP- were subjected to N2a cells and regularly passaged every 7 days up to P8. The detection of newly generated PrP^res^ was assessed by PK digestion as previously described, and the PrP^res^ seeds isolation using PTA was performed according to our previous protocols.

### Building of MD system and simulation protocol

Two simulations of WT HuPrP(90–231) in complex with Cu(II) were performed in order to resemble experimental conditions either at pH 5.5 or pH 7.0. Based on experimental results, in the simulation resembling pH 5.5 (hereafter termed 2His) both H96 and H111 were bound to Cu(II), while at pH 7.0 (hereafter termed 1His) only H111 was bound to the metal. Starting coordinates of HuPrP(90–231) were obtained from the 1QM1 PDB structure^4^. Missing hydrogen atoms were added by MolProbity^38^ and protonation states counter-checked with the H^++^ code^39^. To model the binding of Cu(II) in the active site a set of harmonic springs was used to enforce copper-ligand distances^40^. Bond lengths of the springs were based on EXAFS data and set to 1.98 Å for the N_δ_ imidazole atoms in H96 and H111, to 1.98 Å for the amide oxygen in Q98 and to 3.25 Å for the S atom in M109. The systems were modeled using the AMBER99SB-ILDN* all-atom force field^41^ implemented in the GROMACS MD package version 4.6.5^42^. The protein was immersed in a rhombic dodecahedron box with a minimum distance of 2.5 nm from the box edges; a long distance from the box edges was used to take into proper account the flexibility of HuPrP(90–231). The resulting system was composed of 2192 protein atoms, 54873 TIP3P^43^ water molecules, 1 Cl^−^ ion for a total of 166812 atoms. Electrostatic interactions were accounted by means of the Particle Mesh Ewald method (PME) using a cutoff of 1.5 nm for the real space and Van der Waals interactions^44^. The LINCS algorithm was used to constrain bond lengths and angles^45^, with the exception of Cu(II)-protein bonds. Relaxation of solvent molecules and Cl^−^ ion was initially performed keeping solute atoms restrained to their initial positions with a force constant of 1000 kJ/(mol • nm^2^), for 3.0 ns in a NPT ensemble and using an integration time step of 1.0 fs. Then, the system was simulated for 5 ns while using a force constant of 1000 kJ/(mol • nm^2^) for bonds involving Cu(II) to force the active site residues in the desired position. At this point H111 was freed to create the 1His system and after this step two separate simulations were carried out. The two systems were carried again to 0 K, the force constants in the active site changed to 100 kJ/(mol • nm^2^) and then heated again to 298.15 K. The two systems were then simulated for 150 ns in a NVT ensemble with a time step of 2.0 fs and the neighbor list was updated every 10 steps. Temperature was kept constant using the velocity rescale method with a coupling constant of 0.1 ps during sampling^46^. Structures were clustered using backbone atoms and the GROMOS method^47^. A total of 15000 frames from each simulation were selected with constant pace of 10 ps and a Root Mean Square Deviation (RMSD) threshold of 0.30 nm; only the positions of Cα carbon atoms were used for clustering. Two residues were considered to be bound by a hydrogen (H) bond in a given frame if the H-acceptor distance was below 0.375 nm and the acceptor – donor– H angle was below 40.0°. Two residues are connected by a salt bridge if the nitrogen-oxygen distance is below 0.4 nm in a given frame. All analyses were carried out with standard tools present in the GROMACS MD package v. 4.6.5 or with in-house written codes, except for secondary structure assignment which was performed with DSSP^48^. Figures were produced with Chimera 1.10 software.

## Results

### Copper coordination in the non-OR region of WT HuPrP

**Figure 2:**
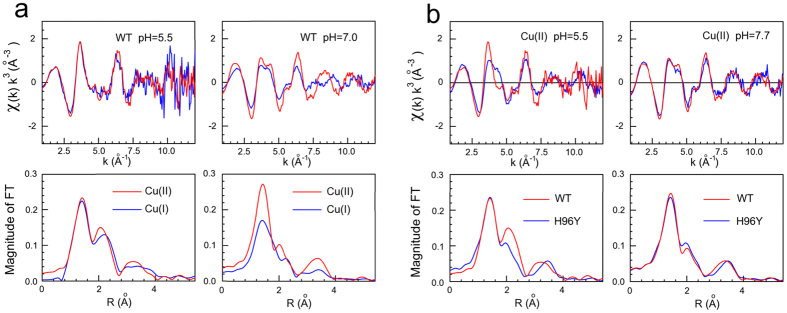
Copper coordination in the non-OR region of WT and H96Y HuPrP. k^3^-weighted EXAFS spectra and Fourier transforms of the experimental data of Cu(II) and Cu(I) bound to WT HuPrP(90–231) at pH 5.5 and 7.0 (**a**), and of Cu(II) bound to WT HuPrP(90–231) and H96Y at pH 5.5 and 7.0 (**b**).

A previous XAS investigation was used to determine the atomic structure of non-OR copper-binding site in the WT HuPrP(90–231)^30^; in this work XAS experiments at the Cu K-edge were carried out at pH 5.5 and the structure of the binding site of both Cu(II) and Cu(I) was found to be identical. Quantitative analysis of the EXAFS spectra indicated that in both oxidation states, copper ion is coordinated by two His residues (H96 and H111 with Cu-N distance of 1.98(2) Å), by two low Z ligands (either oxygen or nitrogen atoms at 1.98 (1.99) Å) and by one sulphur scatterer at longer distance (3.25 Å)^30^. To highlight the effect of pH on the local coordination of the copper ion, XAS spectra of both Cu(II) and Cu(I) HuPrP(90–231) complexes were collected at pH 7.0 (Fig. 2a). While at pH 5.5 the EXAFS spectra are almost identical for both copper oxidation states, at pH 7.0 EXAFS signals show markedly different features over the full *k*-range. The quantitative analysis of the EXAFS data indicates that at pH 7.0 only a single His coordinates the metal ion in both oxidation states, thus suggesting that one of the two His residues (H96 or H111) moves away from the metal. EXAFS data concerning Cu(II)-HuPrP(90–231) at pH 7.0 could be modeled as a four coordinate copper center with one His at 1.99(2) Å, and three N/O scatterers at 1.99(4) Å with a more distant sulphur scatterers at 3.47(4) Å. As far as the Cu(I)-HuPrP(90–231) protein is concerned, the EXAFS analysis revealed a three-fold coordination with one His at 1.98(2) Å, one N/O scatterers at 2.00(2) Å and one sulphur scatterer at 2.27(4) Å (Supplementary Fig. 1). In this case one Met residue enters the Cu(I) first coordination shell, similarly to what previously found at acidic pH value^30^.

### Copper coordination in H96Y, P102L and Q212P HuPrP mutants

**Figure 3:**
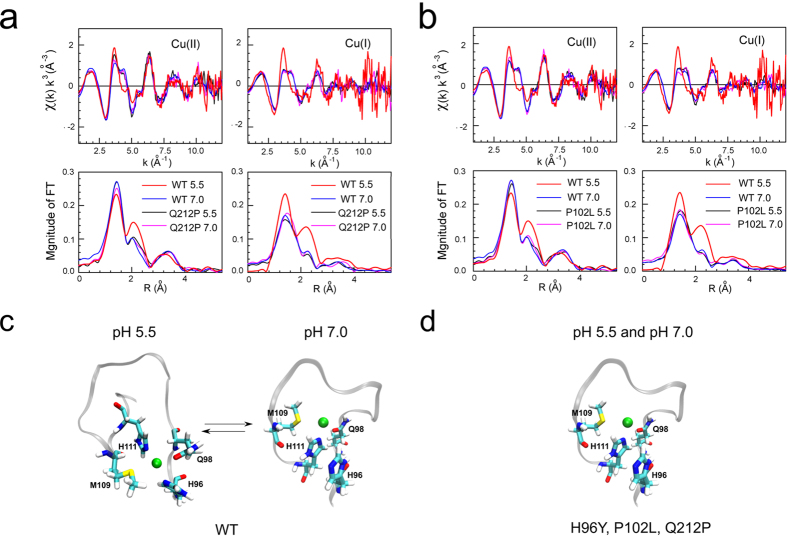
Comparison between copper K-edge XAS experimental data of pathological Q212P, P102L and WT HuPrP. k^3^-weighted EXAFS spectra and Fourier transforms of Cu(II) and Cu(I) bound to WT HuPrP(90–231) and Q212P at pH 5.5 and 7.0 (**a**) and of Cu(II) and Cu(I) bound to WT HuPrP(90–231) and P102L at pH 5.5 and 7.0 (**b**). Schematic representations of copper binding sites in the WT HuPrP(90–231) (**c**) and in the mutants (**d**) at both pH 5.5 and 7.0. Green spheres identify a single cupper ion at both oxidative states.

The Cu(II) local coordination structure in the H96Y mutant was investigated both at pH 5.5 and 7.0 values. At pH 5.5, the Cu K-edge EXAFS data for H96Y, compared to the WT protein, exhibited a clear modification in the coordination environment. This variation is explained by the existence of a single His in the non-OR region of the H96Y mutant, hampering the coordination of the Cu(II) ion with two His residues (Fig. 2b). Conversely, at pH 7.0 the EXAFS spectra of the H96Y mutant and WT proteins are almost identical, thus suggesting that H111 is involved in the copper binding site in both cases. In addition, the EXAFS data showed that the H96Y mutant maintains the same coordination environment around the Cu(II) ion when increasing pH from 5.5 to 7.0. The analysis of the EXAFS data revealed the presence of a four coordinate copper center almost identical to that of the WT protein at pH 7.0, with the H111 residue at 2.00 (2) Å from the ion (Supplementary Table 1). The structures of Cu(II) and Cu(I) binding sites were also investigated in Q212P and P102L HuPrP mutants at both pH 5.5 and 7.0 values. The EXAFS data of the mutants share the same coordination pattern observed for the WT at pH 7.0, where the copper ion is coordinated by a single His, namely H111 (Fig. 3a,b and Supplementary Table 1). In conclusion, while in the WT the copper ion -at both oxidative states- changes coordination losing the contact with H96 at pH 7.0 (Fig. 3c), in the mutants copper is bound only to H111 independently of the pH (Fig. 3d).

### The H96Y mutation promotes prion conversion in neuroblastoma cells

**Figure 4:**
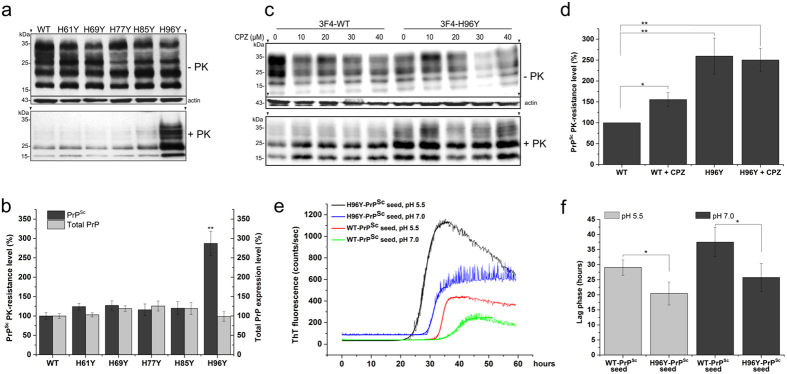
The non-OR H96Y mutation promotes prion conversion in ScN2a cells. (**a**) Fifty μg of undigested lysates from ScN2a cells expressing 3F4-tagged WT and mutated MoPrPs was applied to each lane. Five hundred μg of cell lysate was digested with PK (20 μg/mL) at 37 °C for 1 hour. MoPrPs were detected by anti-PrP 3F4 antibody. β-actin was used as internal loading control. Arrows (▾) indicate positions on the gels where the blots have been cropped. Full-length blots are presented in the Supplementary Figure S4a–b. (**b**) Quantitative analysis of total PrP expression and PrP^Sc^ PK-resistance levels in transfected constructs (n = 4, **P < 0.005, by two-tailed *t* test). (**c**) Copper chelation promoted increased PrP^Sc^ formation in 3F4-WT transfected ScN2a cells. CPZ, cuprizone. Full-length blots are presented in the Supplementary Figure S4c–e. (**d**) Quantitative analysis of PrP^Sc^ PK-resistance levels in 3F4-WT and 3F4-H96Y MoPrP transfected ScN2a cells treated with 10 μM CPZ (n = 3, **P < 0.005 and *P < 0.05). (**e**) ASA showing the kinetics of MoPrP fibrillization in the presence WT and H96Y-PrP^Sc^ seeds at pH 5.5 and 7.0 and (**f**) the corresponding mean value of the lag phases in hours (n = 4, *P < 0.05).

The observed structural differences in the copper coordination among WT and pathological mutants at pH 5.5 may have relevant physiological implications since this alteration in the copper binding site might trigger PrP^C^ to PrP^Sc^ conversion. Hence, the non-OR region could be an important “hot spot” for prion conversion. We first investigated the effect on prion replication of single His residues along the entire N-terminal PrP^C^ domain. ScN2a cells were transiently transfected with 3F4-tagged WT and mutant MoPrP constructs in which each individual His located inside the OR and non-OR copper binding sites were substituted by Tyr, thus abolishing the physiological copper binding. The introduction of the 3F4-epitope tag into these constructs makes it possible to discriminate between transfected and endogenous PrP^C^. His to Tyr substitutions in MoPrP did not affect the total PrP expression levels (Fig. 4a,b and Supplementary Fig. 2a). The enhanced resistance to protease digestion is a primary feature to discriminate between PrP^Sc^ and PrP^C^ in cells chronically infected by prions. The PK digestion profiles showed remarkably different PrP^Sc^ levels among the mutants. While other mutants in the OR region displayed negligible PK-resistant PrP^Sc^ levels similar to the WT, the H96Y mutant yielded a significantly higher PK-resistant PrP^Sc^ signal, providing a first evidence for the role of H96Y mutation in prion conversion (Fig. 4a,b).

Because H96 binds copper, it is likely that the removal of this copper ligand might render PrP^C^ more prone to the conversion in ScN2a cells. Consequently, the absence of copper from the non-OR region could promote this pathological process. To verify this hypothesis, we measured the PrP^Sc^ PK-resistance levels in ScN2a cells transfected either with WT or H96Y MoPrP and treated for 48 hours with cuprizone (CPZ), a well known selective Cu(II) chelator that does not affect cell viability and cannot cross plasma membranes^49^. CPZ treatments on WT ScN2a cells promoted a significantly increase of PrP^Sc^-PK resistance levels upon 10 to 40 μM CPZ additions, suggesting that PrP^C^ in the apo form is more susceptible to PrP^Sc^ conversion (Fig. 4c,d and Supplementary Fig. 2b). The PrP^Sc^ levels remained a plateau among control and CPZ-treated H96Y ScN2a cells (Fig. 4c,d) but always higher than PrP^Sc^ level in the WT cells as also previously presented (Fig. 4a,b). These data are consistent with the hypothesized mechanism whereby H96Y mutant is *per se* sufficient to generate high amount of PrP^Sc^ molecules. Copper appeared as a pivotal modulator of this process since its absence from PrP^C^ side seems to promote prion conversion.

### Biochemical properties of H96Y mutant

We then investigated whether purified PrP^Sc^ from H96Y ScN2a cells shares biochemical properties typical of natural prions, including features such as templating the β-sheet conversion of new PrP molecules, partially PK-resistance, positivity to ThT and ThS staining, cell-to cell transmissibility and intracellular accumulation. We therefore evaluated these properties *in vitro* and in cell-based experiments.

**Figure 5:**
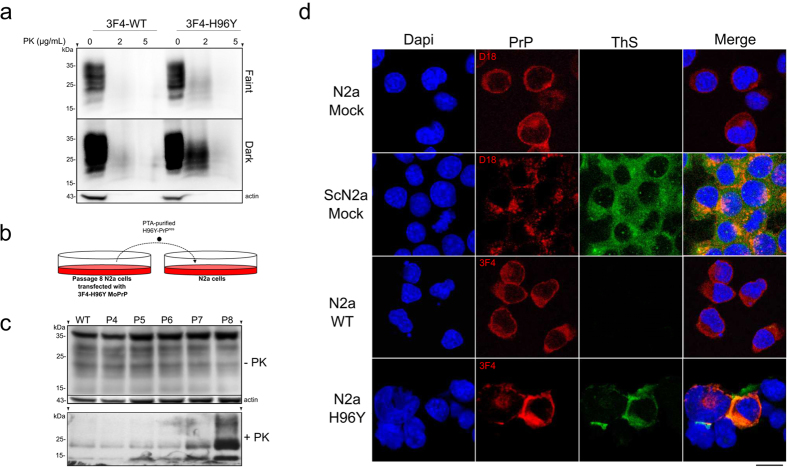
Biochemical properties and cellular localization of H96Y mutant in N2a cells. (**a**) The H96Y mutant displays mild PK-resistance when expressed in N2a cells regularly passaged every 7 days up to passage (P) 8. Cell lysates were treated with 2 or 5 μg/mL of PK. Two exposures of the same blot are shown (Faint: 30 sec exposure; Dark: 6 min exposure). PrPs were detected by anti-PrP 3F4 antibody. β-actin is used as internal control. Arrows (▾) indicate positions on the gels where the blots have been cropped. Full-length blots are presented in the Supplementary Figure S5a–b. (**b**) Schematic representation of the seeding experiment in N2a cells. (**c**) PTA-extracted PrP^Sc^ from N2a cells transfected with 3F4-H96Y MoPrP were inoculated into N2a cells and regularly passaged every 7 days up to P8. The PrP^res^ detection was assessed by PK digestion (5 μg/mL) through passages. Full-length blots are presented in the Supplementary Figure S5c–d. (**d**) ThS-positive H96Y MoPrP aggregates detected in N2a cells. The cells were stained for PrP expression (red) and ThS (green). Untransfected N2a and ScN2a cells (mock) were used as controls. Scale bar: 12 μm.

By means of amyloid seeding assay, PTA-isolated PrP^Sc^ seeds from ScN2a cells expressing H96Y mutant were used to promote the conversion of recombinant full-length WT MoPrP. Differently from the WT-PrP^Sc^ seed, we found that the addition of the H96Y-PrP^Sc^ seed significantly promoted MoPrP fibrillization reactions at both pH 5.5 and 7.0 values, resulting in ThT positive kinetics with shorter lag-phases than the controls (Fig. 4e,f). To test the hypothesis that H96Y mutant causes prion formation in non-prion infected cells, N2a cells were transfected with 3F4-WT or H96Y mutant MoPrP and regularly passaged. We found immunoreactive mildly PK-resistant PrP bands –denoted as PrP^res^ or *bona fide* PrP^Sc^ in the absence of *in vivo* assays- starting from passage (P) 4 to P8 (Fig. 5a and Supplementary Fig. 2c). The isolated H96Y-PrP^res^ material observed in P8 was then used as infectious seed in new N2a cells regularly passaged up to P8 (Fig. 5b). Interestingly, we observed an increment in PrP^res^ levels over passages, thus indicating that H96Y-PrP^res^ seeds induced *de novo* conversion of endogenous PrP^C^ to PK-resistant PrP material (Fig. 5c). Subsequently, we evaluated whether N2a cells transfected with H96Y mutant displayed tinctorial features reminiscent of PrP^Sc^. By using Thioflavin-S (ThS), a specific dye for staining in cells the protein aggregates enriched in amyloid motifs, we found ThS-positive cytoplasmic H96Y mutant deposits similarly to ScN2a (Fig. 5d).

**Figure 6:**
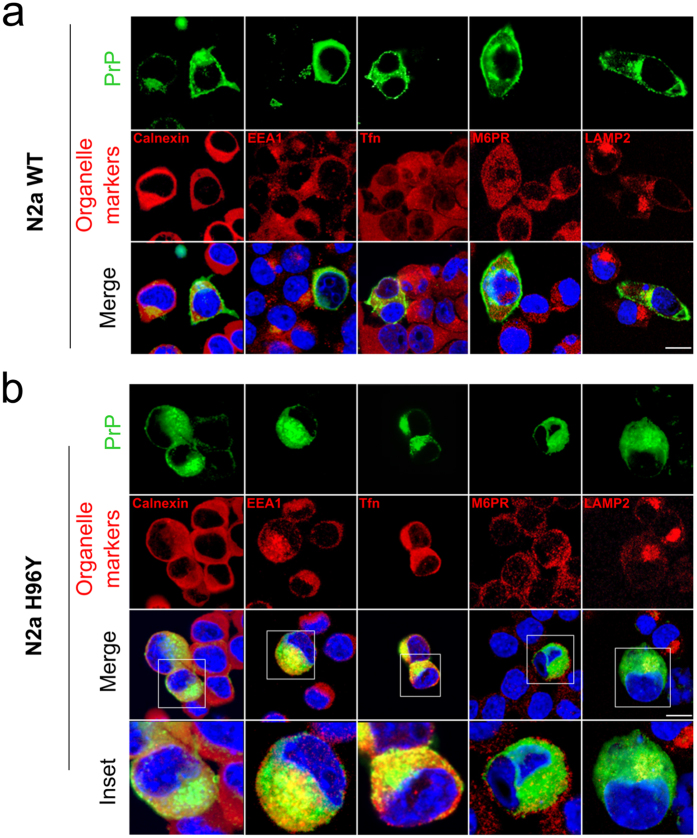
The H96Y MoPrP mutant displays intracellular accumulation in the endosomial compartments. PrP localization in N2a cells expressing the 3F4-WT MoPrP (**a**) or H96Y MoPrP (**b**). Nuclei are labeled with DAPI (blue), PrPs are detected by 3F4 antibody (green); organelle markers, such as Calnexin (ER marker), EEA1 (early endosomes marker), Tfn (recycling endosome marker), M6PR (late endosome marker) and LAMP2 (lysosome marker) are labeled in red. Insets in (**b**) shows a magnification of the merged panels (white boxed areas). Scale bars: 12 μm.

The observation that the H96Y mutant forms ThS-positive aggregates demands the identification of the primary intracellular compartments where H96Y mutant accumulation occurs. Consistent with previous studies, 3F4-WT MoPrP was found mostly on the cell surface and it was detectable along the ER, endosomal and lysosomal compartments. The WT and H96Y showed co-localization with the ER marker calnexin indicating a correct trafficking through the ER (Fig. 6a). Biochemical analysis on transfected WT and mutant MoPrP showed that the proteins display the same glycosylation patterns and molecular weight after Endo-H and PNGase-F treatments (Supplementary Fig. 2d). However, we found a significant population of H96Y mutant co-localizing with organelle markers as EEA1 (early endosomes), Tfn (recycling endosomes), M6PR (late endosome) and LAMP2 (lysosome marker) (Fig. 6b), suggesting a predominant accumulation of the mutant in the acidic compartments, as reported in previous studies on PrP^Sc^ intracellular accumulation.

### Removal of the H96-Cu(II) bond creates transient N-terminal β-sheet structures

To interpret our results from a structural biology point of view, we performed MD simulation studies to predict the structural facets of WT HuPrP(90–231) coordinating Cu(II) either with His96 and His111 or with His111, termed 2His and 1His models, respectively.

**Figure 7:**
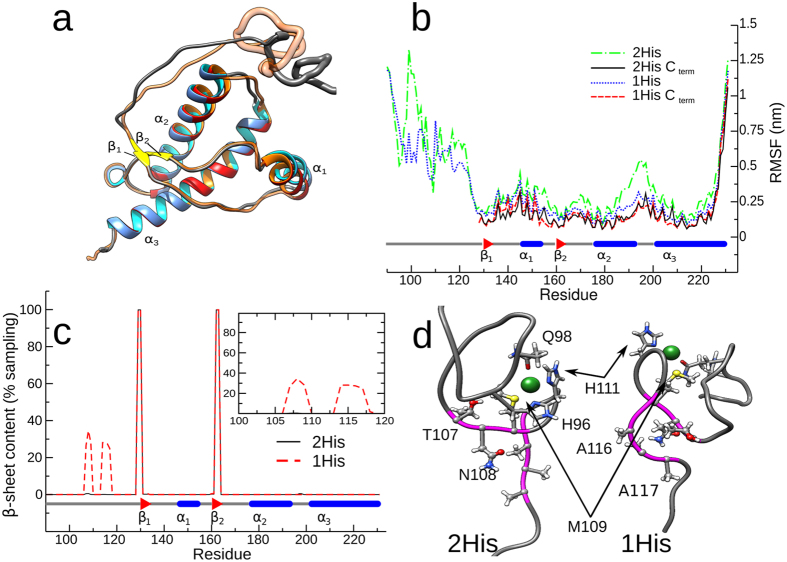
The ion coordination sphere affects HuPrP(90–231) dynamics in MD simulations. (**a**) Superimposition of average structures sampled in the 2His and 1His trajectories. Helical domains are represented in blue and red in 2His and 1His, respectively, the β_1_ and β_2_ sheets are in yellow, coils are represented in gray (2His) and orange (1His); the non-OR regions are depicted using a wider transparent ribbon. (**b**) Root Mean Square Fluctuation (RMSF) of residues for the 2His and 1His simulations. RMSF was calculated fitting the coordinates of the complete protein (2His: green dot-dashed line; 1His: blue dotted line) or restricted to the C-terminal domain (2His: black line; 1His: red dashed line). In both simulations the N-terminal domain is, as expected, much more flexible with the exception of residues 226–231; the 2His simulation apparently features greater fluctuations but the differences fade out when only the stable C terminal domain is considered. Notable exceptions are residues H140 and R151. (**c**) Residue-wise β-sheet content for the 2His and 1His simulation as a percentage of sampling (150.0 ns); the 1His simulation shows unstable β-sheet formation between residues 106–109 and 114–117 (also shown in the inset). (**d**) Comparison of the first centroid obtained by clustering the N-terminal domain Cα atoms in the 2His (left) and 1His (right) simulations; residues 106–109 and 114–117 are shown in ball and stick (backbone is shown in magenta as in Supplementary Fig 3a); copper binding residues H96, Q98 and H111 are shown as sticks and the copper ion as a dark green sphere (note that M109 belongs to both groups); a number of residues forming hydrogen bonds unique to one system are explicitly labeled to improve clarity.

The removal of H96 from the Cu(II) coordination sphere did not produce significant changes in the globular domain (residues 128–231), showing similar α_2_-α_3_ helix orientation as suggested by previous MD and NMR studies^4^,^50^ (*e.g.* α_2_-α_3_ helix angle is 51.2° and 49.1° in 2His and 1His models, respectively, while it is 50.9° in NMR structure) (Fig. 7a). While the C-terminal domain in the two simulation models featured a comparable flexibility (residue-wise Root Mean Square Fluctuation), the N-terminal segment of the 2His model displayed higher flexibility, particularly between residues 98–102 and 116–123 (Fig. 7b and Supplementary Fig. 3a). Additionally, the 2His trajectory yielded a slightly higher radius of gyration as compared to the 1His (1.77 ± 0.08 nm *vs* 1.74 ± 0.04 nm) with more pronounced oscillations (Supplementary Fig. 3b) confirming that the N-terminal segment in the 2His trajectory was relatively more disordered. The reduced flexibility of the residues 90–120 segment observed in the 1His trajectory is attributed to the presence of novel hydrogen (H)-bonds and salt bridges, which were uniquely present in the 1His simulation. In particular, we observed that in the 2His system the side-chain of T95 formed H-bond in 89% of sampling with the backbone oxygen of T107, the backbones of T95 and P105 formed a H-bond for 92% of sampling and the side-chains nitrogen atom of Q98 (a residue involved in copper binding) and N100 formed a H-bond in 51% of sampling. In the 1His trajectory, the backbones of residues N108 and A116 formed H-bond in 58% of sampling and the oxydryl group of T107 bound to the backbone of P105 in 62% of sampling. These alteration of the internal hydrogen bond network in the 1His system created favorable conditions for transient β-sheet motif formations in the segments formed by residues 106–109 and 114–117 (with a lifetime between 20% and 30% of sampling) which were not observed in the 2His trajectory (Fig. 7c and Supplementary Fig. 3a). A cluster analysis was performed on the N-terminal domain (residues 90–121) of the 2His and 1His trajectories, using 15000 even spaced configurations. More than 80% of the configurations in both trajectories were included in the first cluster (12017 for 2His and 13347 for 1His, respectively). Comparison of the first centroid structures obtained from the 1His and 2His simulations (Fig. 7d) shows that in the former case these groups of residues are indeed roughly antiparallel, with the side-chains of residues 106–109 oriented towards the backbone of residues 114–117, *i.e.* in a favorable position for the formation of a small β-sheet. On the other hand, in the 2His trajectory these two clusters form an angle of approximately 90° in most of the sampled structures.

## Discussion

The central molecular event in prion diseases is the conversion of PrP^C^ into pathological and infectious prions. Despite numerous investigations, the conversion mechanism(s) leading to PrP^Sc^ formation and transmission remain unclear. NMR-based studies on both Hu and MoPrP structured domains have proposed a role of the β_2_-α_2_ loop as dynamic “switch” element able to modulate prion conversion and susceptibility of a given specie to TSE^51^. However, the structural rearrangements occurring at the N-terminal region have not yet been clarified due to its intrinsic disorder. In a previous X-ray crystallography study we found that the palindromic domain can initiate β-sheet enrichment when HuPrP is crystallized in complex with a Nanobody^24^, suggesting that the segment 90–127 may act as alternative N-terminal switch for prion conversion. This region attracted interest because of the ability of the palindromic motif to form neurotoxic species^52^ and the proximity of the non-OR region, arguing a possible pathological link between copper binding to this site and prion conversion.

Here, we provide structural and biological evidence that the non-OR region may have pivotal biological implication for prion formation. We found that mutations –*i.e.* the GSS-causing Q212P and P102L mutants and the artificial H96Y mutation- and pH exchanges cause a dramatic modification on both Cu(II) and Cu(I) coordinations in the non-OR region. In the WT HuPrP Cu(II) and Cu(I) are anchored to His96 and His111 only at pH 5.5, while at pH 7 copper at both oxidative states is coordinated by His111. Conversely, in the mutants copper is bound only to H111 independently of the pH. The MD simulations propose a model whereby the molecular switch between the 2His and 1His systems (*i.e.* copper bound to H96 and H111, or only to H111, respectively) may have structural implications. The 1His coordination seems to favor β-sheet enrichment in the region encompassing residues 106–117. At this stage it is unclear how this early β-sheet conversion may drive a complete structural conversion to PrP^Sc^, but the new structural rearrangements occurring at the palidromic motif might serve as a nucleus for the association of intermolecular β-strands.

To interpret these structural clues in the context of more relevant physiological implications, we used ScN2a and N2a cells transiently expressing 3F4-MoPrP with the H96Y mutation. This construct mimics the pathological P102L and Q212P mutants investigated by EXFAS and it may represent a useful model to elucidate the role of the non-OR region for prion conversion. His to Tyr96 substitution removes one crucial copper ligand, thus it may allow to link the effect caused by altered copper coordination with prion conversion. The expression of H96Y mutant highly promotes prion conversion in ScN2a cells and spontaneously generated PrP^res^ together with intracellular MoPrP accumulation in N2a cells. We hypothesize that the non-OR copper-binding site at H96 is much more important for both PrP^C^ function, as shown by the higher affinity for copper binding compared to the OR region^53^, and prion propagation and infectivity^54^. Residue H96 is located in a PrP^Sc^ region that is partially resistant to PK, thus copper bound to H96 may have a role during PrP^Sc^ formation even in the absence of the OR region^55^. On the basis of our findings, we propose that the H96Y mutant may act as other pathogenic mutations located in both N- and C-terminal domains (*e.g.* P102L and Q212P), causing spontaneous prion conversion. Our analysis shows that substitution of H96Y mutation induces PrP^C^ accumulation in the acidic endosomal compartments, as observed for other disease-linked mutations^56^. The pH change from neutral to acidic values has been proposed to trigger the PrP^C^ conformational conversion and a change in the balance of distinct internalization mechanisms may promote PrP^Sc^ replication by diverting the protein to distinct intracellular compartments and inhibiting the cellular protein quality control systems^57^. We report here enhanced PK-resistance of the H96Y mutant when expressed in ScN2a cells and its ability to generate *de novo* PrP^res^ in N2a cells. These findings propose a pivotal role for non-OR region as critical molecular switch for prion conversion. We therefore argue that copper bound to the non-OR region may stabilize this segment when coordinated by His96 and His111, preventing misfolding events through transient short and long range interaction contacts between the 90–127 residues and the C-terminal structured domain. Copper acts as a key modulator of this process since its absence from PrP^C^ side promote prion conversion, as observed in WT ScN2a cells treated with a copper chelator.

**Figure 8:**
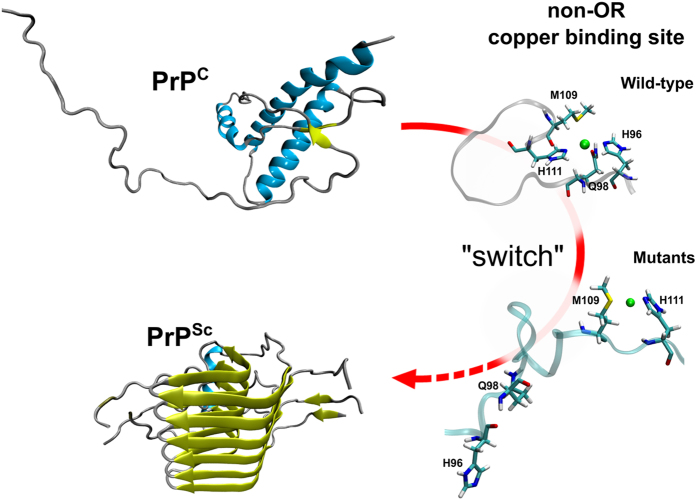
Model for the non-OR region molecular switch at acidic pH. PrP^C^ coordinating copper with one His residue in the non-OR region is more prone to the conversion at acidic pH condition. We propose a model where His96 and His111 represent the N-terminal switch for prion conversion in the PrP^C^. As PrP^Sc^ model we used the HET-s(218–289) 2KJ3 PDB structure.

Our study highlights the importance of the non-OR region for prion conversion and suggests a model in which PrP^C^ coordinating copper with one His may be more prone to the conversion at acidic condition (Fig. 8). Thus, the non-OR region may act as the long-sought-after prion switch. The data presented here provide the bases for experiments aimed at elucidating whether the H96Y mutation causes TSE when expressed in animal models.

## Additional Information

**How to cite this article**: Giachin, G. *et al.* The non-octarepeat copper binding site of the prion protein is a key regulator of prion conversion. *Sci. Rep.* **5**, 15253; doi: 10.1038/srep15253 (2015).

## Supplementary Material

Supplementary Information

## Figures and Tables

**Figure 1:**
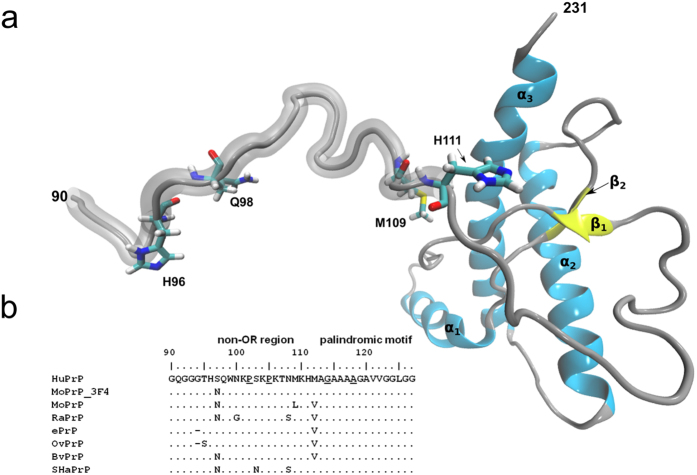
The non-OR region in the truncated HuPrP. In (**a**) cartoon representation of the HuPrP(90–231) with highlighted the segment 90–111 including the apo form of the non-OR copper binding site. Residues H96, Q98, M109 and H111 contribute to coordinate one Cu(II) or Cu(I) ion. In (**b**), alignment of residues 90–127 including the non-OR region and the palindromic motif of different mammalian prion proteins (PrP): HuPrP (human, *Homo sapiens*, NCBI accession code AAA60182), MoPrP (mouse, *Mus musculus*, AAA39997) and MoPrP_3F4 (carrying the 3F4 tag), RaPrP (rabbit, *Oryctolagus cuniculus*, AAD01554.1), ePrP (elk, *Cervus elaphus nelsoni*, AAB94788), OvPrP (sheep, *Ovis aries*, AFM91142.1), BvPrP (bank vole, *Myodes glareolus*, AAL57231) and SHaPrP (Syrian hamster, *Mesocricetus auratus*, AAA37091). In the HuPrP primary sequence the residues involved in GSS-linked mutations are underlined (P102L, P105L, G114V and A117V).

## References

[b1] Colby D. W. & Prusiner S. B. Prions. Cold Spring Harb Perspect Biol 3, a006833 (2011). 21421910 10.1101/cshperspect.a006833 PMC3003464

[b2] Legname G., Giachin G. & Benetti F. Structural Studies of Prion Proteins and Prions. In: Non-fibrillar Amyloidogenic Protein Assemblies - Common Cytotoxins Underlying Degenerative Diseases (ed^(eds Rahimi F. & Bitan G.) Springer: Netherlands, (2012).

[b3] Surewicz W. K. & Apostol M. I. Prion protein and its conformational conversion: a structural perspective. Top Curr Chem 305, 135–167 (2011). 21630136 10.1007/128_2011_165

[b4] Zahn R. *et al.* NMR solution structure of the human prion protein. Proc Natl Acad Sci USA 97, 145–150 (2000). 10618385 10.1073/pnas.97.1.145 PMC26630

[b5] Walter E. D., Chattopadhyay M. & Millhauser G. L. The affinity of copper binding to the prion protein octarepeat domain: evidence for negative cooperativity. Biochemistry 45, 13083–13092 (2006). 17059225 10.1021/bi060948r PMC2905157

[b6] Walter E. D. *et al.* Copper binding extrinsic to the octarepeat region in the prion protein. Curr Protein Pept Sci 10, 529–535 (2009). 19538144 10.2174/138920309789352056 PMC2905140

[b7] Jobling M. F. *et al.* The hydrophobic core sequence modulates the neurotoxic and secondary structure properties of the prion peptide 106-126. J Neurochem 73, 1557–1565 (1999). 10501201 10.1046/j.1471-4159.1999.0731557.x

[b8] Gong B. *et al.* Probing structural differences between PrP(C) and PrP(Sc) by surface nitration and acetylation: evidence of conformational change in the C-terminus. Biochemistry 50, 4963–4972 (2011). 21526750 10.1021/bi102073j

[b9] Requena J. R. & Wille H. The structure of the infectious prion protein. Prion 8, 60–66 (2014). 24583975 10.4161/pri.28368 PMC7030906

[b10] Nuvolone M., Aguzzi A. & Heikenwalder M. Cells and prions: a license to replicate. FEBS Lett 583, 2674–2684 (2009). 19527722 10.1016/j.febslet.2009.06.014

[b11] Herms J. *et al.* Evidence of presynaptic location and function of the prion protein. J Neurosci 19, 8866–8875 (1999). 10516306 10.1523/JNEUROSCI.19-20-08866.1999 PMC6782778

[b12] Steele A. D., Emsley J. G., Ozdinler P. H., Lindquist S. & Macklis J. D. Prion protein (PrPc) positively regulates neural precursor proliferation during developmental and adult mammalian neurogenesis. Proc Natl Acad Sci USA 103, 3416–3421 (2006). 16492732 10.1073/pnas.0511290103 PMC1413927

[b13] Caiati M. D. *et al.* PrP^C^ controls via protein kinase A the direction of synaptic plasticity in the immature hippocampus. J Neurosci 33, 2973–2983 (2013). 23407955 10.1523/JNEUROSCI.4149-12.2013 PMC6619229

[b14] Santuccione A., Sytnyk V., Leshchyns’ka I. & Schachner M. Prion protein recruits its neuronal receptor NCAM to lipid rafts to activate p59fyn and to enhance neurite outgrowth. J Cell Biol 169, 341–354 (2005). 15851519 10.1083/jcb.200409127 PMC2171870

[b15] Gasperini L., Meneghetti E., Pastore B., Benetti F. & Legname G. Prion Protein and Copper Cooperatively Protect Neurons by Modulating NMDA Receptor Through S-nitrosylation. Antioxidants & Redox Signaling 22, 772–784 (2014). 25490055 10.1089/ars.2014.6032 PMC4361008

[b16] Khosravani H. *et al.* Prion protein attenuates excitotoxicity by inhibiting NMDA receptors. J Cell Biol 181, 551–565 (2008). 18443219 10.1083/jcb.200711002 PMC2364707

[b17] Stys P. K., You H. & Zamponi G. W. Copper-dependent regulation of NMDA receptors by cellular prion protein: implications for neurodegenerative disorders. J Physiol 590, 1357–1368 (2012). 22310309 10.1113/jphysiol.2011.225276 PMC3382327

[b18] Pushie M. J. *et al.* Prion protein expression level alters regional copper, iron and zinc content in the mouse brain. Metallomics 3, 206–214 (2011). 21264406 10.1039/c0mt00037j

[b19] Brown D. R. Prions and manganese: A maddening beast. Metallomics 3, 229–238 (2011). 21390367 10.1039/c0mt00047g

[b20] Walter E. D., Stevens D. J., Visconte M. P. & Millhauser G. L. The prion protein is a combined zinc and copper binding protein: Zn^2+^ alters the distribution of Cu^2+^ coordination modes. J Am Chem Soc 129, 15440–15441 (2007). 18034490 10.1021/ja077146j PMC2532507

[b21] Kardos J., Kovacs I., Hajos F., Kalman M. & Simonyi M. Nerve endings from rat brain tissue release copper upon depolarization. A possible role in regulating neuronal excitability. Neurosci Lett 103, 139–144 (1989). 2549468 10.1016/0304-3940(89)90565-x

[b22] Vassallo N. & Herms J. Cellular prion protein function in copper homeostasis and redox signalling at the synapse. J Neurochem 86, 538–544 (2003). 12859667 10.1046/j.1471-4159.2003.01882.x

[b23] Fischer M. *et al.* Prion protein (PrP) with amino-proximal deletions restoring susceptibility of PrP knockout mice to scrapie. EMBO J 15, 1255–1264 (1996). 8635458 PMC450028

[b24] Abskharon R. N. *et al.* Probing the N-terminal beta-sheet conversion in the crystal structure of the human prion protein bound to a nanobody. J Am Chem Soc 136, 937–944 (2014). 24400836 10.1021/ja407527p

[b25] Younan N. D. *et al.* Copper(II)-induced secondary structure changes and reduced folding stability of the prion protein. J Mol Biol 410, 369–382 (2011). 21619885 10.1016/j.jmb.2011.05.013

[b26] Migliorini C., Sinicropi A., Kozlowski H., Luczkowski M. & Valensin D. Copper-induced structural propensities of the amyloidogenic region of human prion protein. J Biol Inorg Chem 19, 635–645 (2014). 24737041 10.1007/s00775-014-1132-7

[b27] Baumann F. *et al.* Lethal recessive myelin toxicity of prion protein lacking its central domain. EMBO J 26, 538–547 (2007). 17245436 10.1038/sj.emboj.7601510 PMC1783444

[b28] Li A. *et al.* Neonatal lethality in transgenic mice expressing prion protein with a deletion of residues 105-125. EMBO J 26, 548–558 (2007). 17245437 10.1038/sj.emboj.7601507 PMC1783448

[b29] McDonald A., Pushie M. J., Millhauser G. L. & George G. N. New insights into metal interactions with the prion protein: EXAFS analysis and structure calculations of copper binding to a single octarepeat from the prion protein. J Phys Chem B 117, 13822–13841 (2013). 24102071 10.1021/jp408239h PMC3890359

[b30] D’Angelo P. *et al.* Effects of the pathological Q212P mutation on human prion protein non-octarepeat copper-binding site. Biochemistry 51, 6068–6079 (2012). 22788868 10.1021/bi300233n

[b31] Hasnain S. S. *et al.* XAFS study of the high-affinity copper-binding site of human PrP(91-231) and its low-resolution structure in solution. J Mol Biol 311, 467–473 (2001). 11493001 10.1006/jmbi.2001.4795

[b32] Ilc G. *et al.* NMR structure of the human prion protein with the pathological Q212P mutation reveals unique structural features. PLoS One 5, e11715 (2010). 20661422 10.1371/journal.pone.0011715 PMC2908606

[b33] Casey J. R., Grinstein S. & Orlowski J. Sensors and regulators of intracellular pH. Nat Rev Mol Cell Biol 11, 50–61 (2010). 19997129 10.1038/nrm2820

[b34] Parchi P. *et al.* Different patterns of truncated prion protein fragments correlate with distinct phenotypes in P102L Gerstmann-Straussler-Scheinker disease. Proc Natl Acad Sci USA 95, 8322–8327 (1998). 9653185 10.1073/pnas.95.14.8322 PMC20974

[b35] Proux O. *et al.* FAME: A new beamline for X-ray absorption investigations of very-diluted systems of environmental, material and biological interests. Phys Scripta T115, 970–973 (2005).

[b36] Filipponi A., Di Cicco A. & Natoli C. R. X-ray-absorption spectroscopy and n-body distribution functions in condensed matter. I. Theory. Phys Rev B Condens Matter 52, 15122–15134 (1995). 9980866 10.1103/physrevb.52.15122

[b37] Filipponi A. & Di Cicco A. X-ray-absorption spectroscopy and n-body distribution functions in condensed matter. II. Data analysis and applications. Phys Rev B Condens Matter 52, 15135–15149 (1995). 9980867 10.1103/physrevb.52.15135

[b38] Chen V. B. *et al.* MolProbity: all-atom structure validation for macromolecular crystallography. Acta Crystallogr D Biol Crystallogr 66, 12–21 (2010). 20057044 10.1107/S0907444909042073 PMC2803126

[b39] Gordon J. C. *et al.* H++: a server for estimating pKas and adding missing hydrogens to macromolecules. Nucleic Acids Res 33, W368–371 (2005). 15980491 10.1093/nar/gki464 PMC1160225

[b40] Rose F., Hodak M. & Bernholc J. Mechanism of copper(II)-induced misfolding of Parkinson’s disease protein. Sci Rep 1, 11 (2011). 22355530 10.1038/srep00011 PMC3216499

[b41] Aliev A. E. *et al.* Motional timescale predictions by molecular dynamics simulations: case study using proline and hydroxyproline sidechain dynamics. Proteins 82, 195–215 (2014). 23818175 10.1002/prot.24350 PMC4282583

[b42] Van Der Spoel D. *et al.* GROMACS: fast, flexible, and free. J Comput Chem 26, 1701–1718 (2005). 16211538 10.1002/jcc.20291

[b43] Jorgensen W. L., Chandrasekhar J., Madura J. D., Impey R. W. & Klein M. L. Comparison of Simple Potential Functions for Simulating Liquid Water. Journal of Chemical Physics 79, 926–935 (1983).

[b44] Cheatham T. E., Miller J. L., Fox T., Darden T. A. & Kollman P. A. Molecular-Dynamics Simulations on Solvated Biomolecular Systems - the Particle Mesh Ewald Method Leads to Stable Trajectories of DNA, Rna, and Proteins. Journal of the American Chemical Society 117, 4193–4194 (1995).

[b45] Hess B., Bekker H., Berendsen H. J. C. & Fraaije J. G. E. M. LINCS: A linear constraint solver for molecular simulations. Journal of Computational Chemistry 18, 1463–1472 (1997).

[b46] Bussi G., Donadio D. & Parrinello M. Canonical sampling through velocity rescaling. J Chem Phys 126, 014101 (2007). 17212484 10.1063/1.2408420

[b47] Torda A. E. & Vangunsteren W. F. Algorithms for Clustering Molecular-Dynamics Configurations. Journal of Computational Chemistry 15, 1331–1340 (1994).

[b48] Kabsch W. & Sander C. Dictionary of protein secondary structure: pattern recognition of hydrogen-bonded and geometrical features. Biopolymers 22, 2577–2637 (1983). 6667333 10.1002/bip.360221211

[b49] Benetti F. *et al.* Cuprizone neurotoxicity, copper deficiency and neurodegeneration. Neurotoxicology 31, 509–517 (2010). 20685220 10.1016/j.neuro.2010.05.008

[b50] Rossetti G., Giachin G., Legname G. & Carloni P. Structural facets of disease-linked human prion protein mutants: a molecular dynamic study. Proteins 78, 3270–3280 (2010). 20806222 10.1002/prot.22834

[b51] Sigurdson C. J. *et al.* A molecular switch controls interspecies prion disease transmission in mice. J Clin Invest 120, 2590–2599 (2010). 20551516 10.1172/JCI42051 PMC2898603

[b52] Jobling M. F. *et al.* Copper and zinc binding modulates the aggregation and neurotoxic properties of the prion peptide PrP106-126. Biochemistry 40, 8073–8084 (2001). 11434776 10.1021/bi0029088

[b53] Nadal R. C., Davies P., Brown D. R. & Viles J. H. Evaluation of copper2+ affinities for the prion protein. Biochemistry 48, 8929–8931 (2009). 19697960 10.1021/bi9011397

[b54] Quaglio E., Chiesa R. & Harris D. A. Copper converts the cellular prion protein into a protease-resistant species that is distinct from the scrapie isoform. J Biol Chem 276, 11432–11438 (2001). 11278539 10.1074/jbc.M009666200

[b55] Cox D. L., Pan J. & Singh R. R. A mechanism for copper inhibition of infectious prion conversion. Biophys J 91, L11–13 (2006). 16698781 10.1529/biophysj.106.083642 PMC1483082

[b56] Ashok A. & Hegde R. S. Selective processing and metabolism of disease-causing mutant prion proteins. PLoS Pathog 5, e1000479 (2009). 19543376 10.1371/journal.ppat.1000479 PMC2691595

[b57] Baskakov I. V., Legname G., Baldwin M. A., Prusiner S. B. & Cohen F. E. Pathway complexity of prion protein assembly into amyloid. J Biol Chem 277, 21140–21148 (2002). 11912192 10.1074/jbc.M111402200

