# Supplementary material for: The non-octarepeat copper binding site of the prion protein is a key regulator of prion conversion
Source: Sci Rep. 2015 Oct 20;5:15253. doi: 10.1038/srep15253 (PMC4651146; doi:10.1038/srep15253)
Supplement: Supplementary Information [file srep15253-s1.doc]

**Supplementary Information**

**The non-octarepeat copper binding site of the prion protein is a key regulator of prion conversion**

Gabriele Giachin^1,2^, Phuong Thao Mai^1,9^, Thanh Hoa Tran^1^, Giulia Salzano^1^, Federico Benetti^1,10^, Valentina Migliorati^3^, Alessandro Arcovito^4^, Stefano Della Longa^5^, Giordano Mancini^6,7^, Paola D’Angelo^3,11^ and Giuseppe Legname^1,8,11^

^1^ Department of Neuroscience, Scuola Internazionale Superiore di Studi Avanzati (SISSA), Trieste, Italy.

^2^ Structural Biology Group, European Synchrotron Radiation Facility (ESRF), Grenoble, France.

^3^ Department of Chemistry, Sapienza University of Rome, Rome, Italy.

^4^ Istituto di Biochimica e Biochimica Clinica, Università Cattolica del Sacro Cuore, Rome, Italy.

^5^ Department of Medicine, Public Health, Life and Environmental Science, University of L'Aquila, Coppito Aquila, Italy.

^6^ Scuola Normale Superiore, Pisa, Italy.

^7^ Istituto Nazionale di Fisica Nucleare (INFN) sezione di Pisa, Pisa, Italy.

^8^ ELETTRA - Sincrotrone Trieste S.C.p.A, Trieste, Italy.

^9^ Present address: University of Medicine and Pharmacy at Ho Chi Minh City, Ho Chi Minh City, Vietnam.

^10^ Present address: European Center for the Sustainable Impact of Nanotechnology, Veneto Nanotech S.C.p.A., Rovigo, Italy.

^11^ Correspondence should be addressed to P.D. ([p.dangelo@uniroma1.it](mailto:p.dangelo@uniroma1.it)) and G.L. ([legname@sissa.it](mailto:legname@sissa.it)).


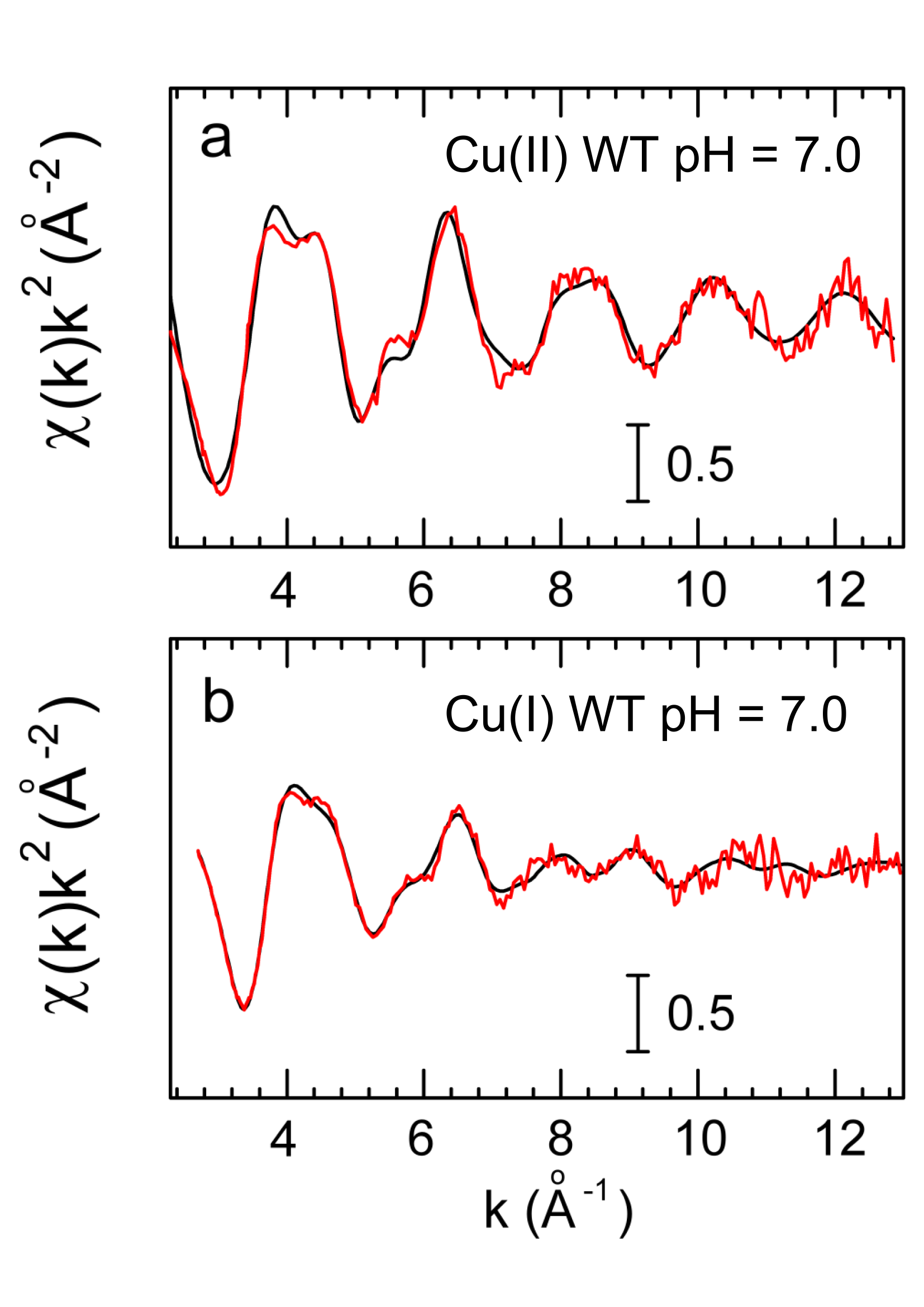


**Figure S1. Analysis of the Cu K-edge X-ray absorption spectra.** (**a**) Cu K-edge EXAFS experimental data (red) and best fit theoretical signal (black) of Cu(II) WT HuPrP(90-231) protein at pH 7.0. The EXAFS fit has been carried out in the *k* range 2.4-13.0 Å^-1^. The Cu(II) ion is coordinated with a single histidine residue with a Cu-N distance of 1.99 Å and with a sulphur atom at 3.37 Å. (**b**) Cu K-edge EXAFS experimental data (red) and best fit theoretical signal (black) of Cu(I) WT HuPrP(90-231) protein at pH 7.0. The EXAFS fit has been carried out in the *k* range 2.5-13.0 Å^-1^. The Cu(I) ion first coordination shell is formed by an histidine residue with a Cu-N distance of 1.99 Å and a methionine residue with a Cu-S distance of 2.27 Å.


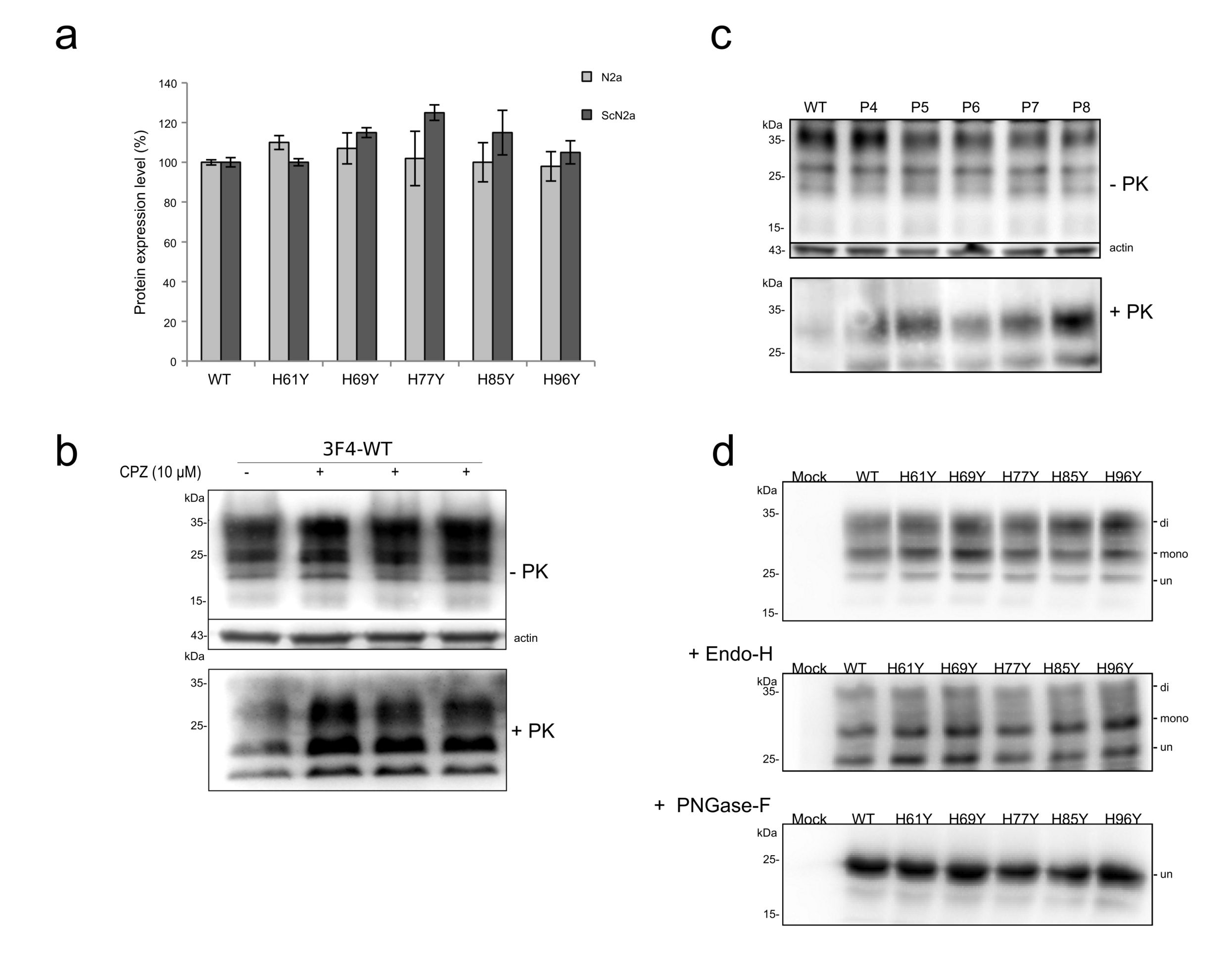


**Figure S2.** (**a**) Quantitative analysis of actin-normalized total PrP expression levels in both N2a and ScN2a cells transfected with 3F4-tagged MoPrP constructs (n = 4). (**b**) Ten μM cuprizone (CPZ) treatments (n=3) on ScN2a cells transfected with 3F4-WT MoPrP promoted increased PrP^Sc^ levels. (**c**) N2a cells were transiently transfected with 3F4-H96Y MoPrP and regularly passaged every 7 days up to passage (P) 8. The PrP^res^ levels were monitored upon PK digestion and detected by anti-PrP 3F4 antibody. β-actin is used as internal control. (**d**) The OR and non-OR mutations share the same glycosylation patterns and proteolytic features when treated with Endo-H and PNGase-F as the WT MoPrP^C^. The positions of diglycosylated, monoglycosylated and unglycosylated forms (denoted as di, mono and un) of PrP^C^ are on the right of each WB. PrPs were detected by anti-PrP 3F4 antibody.


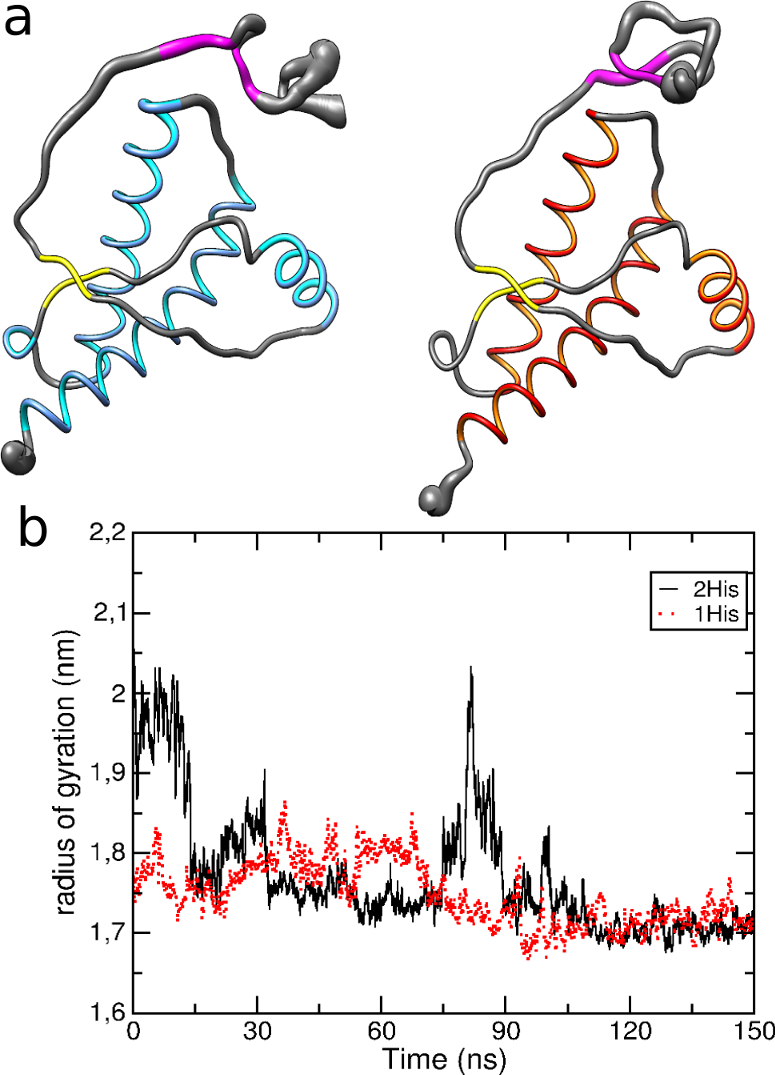


**Figure S3.** (**a**) Cartoon representation of RMSF. Ribbon width is proportional to fluctuations; the coordinates correspond to those of the first centroid obtained for each trajectory by cluster analysis of the C-terminal domain. The ratio between radius and RMSF is the same in the two models. The average relative orientation of residues 106-109 and 114-117 (colored in magenta) in the 1His system is also clearly observable. (**b**) Radius of gyration (*R_g_*) calculated for the 2His (black) and 1His (red) trajectories. The *R_g_* of the 2His trajectory features transitions between different conformation with *R_g_* up to 2 nm with a lifetime of several nanoseconds each while the *R_g_* of the 1His simulated is stably oscillates around 1.74 nm.


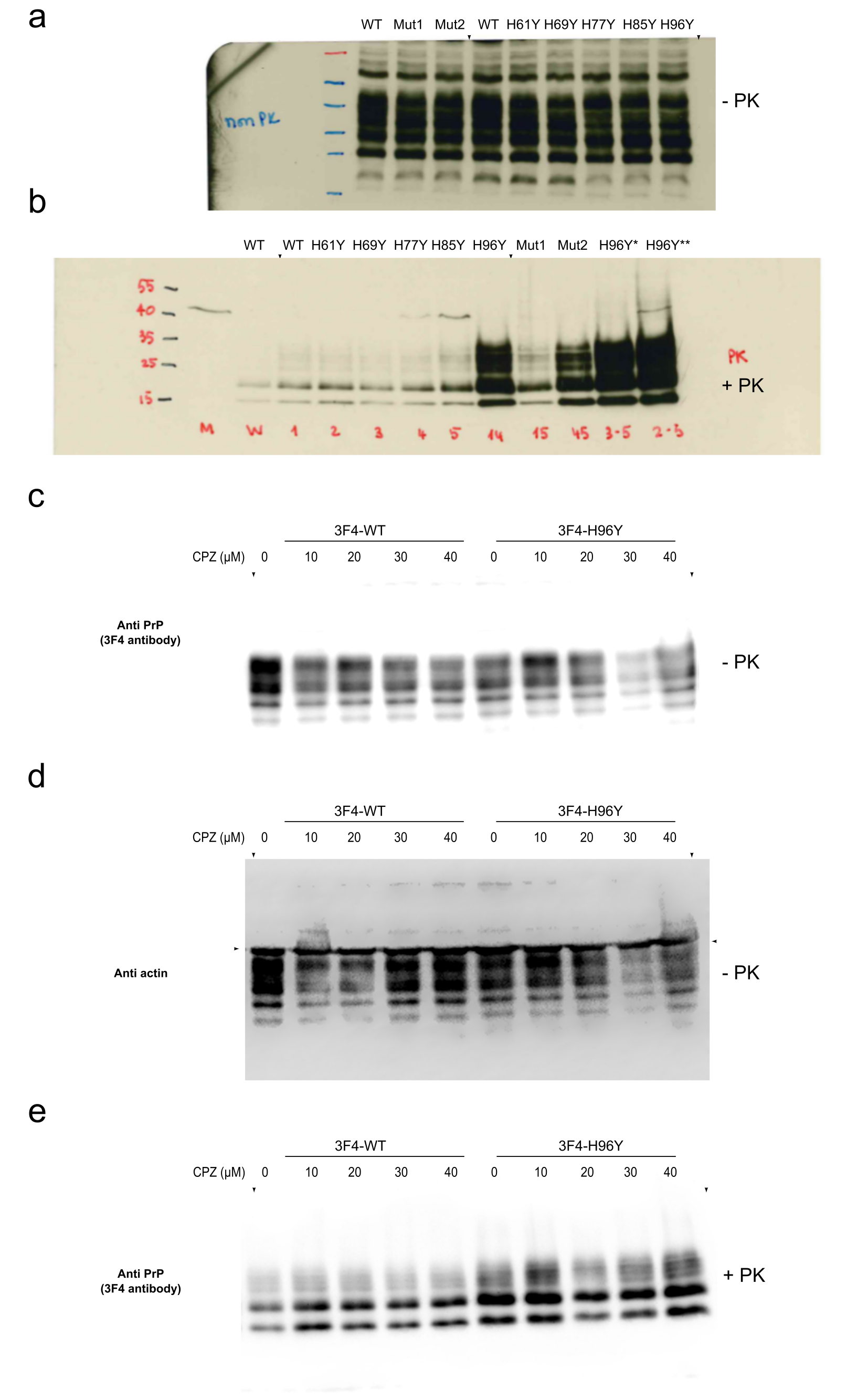


**Figure S4.** Full-length western-blots (WB) presented in Figure 4a and 4c in the main manuscript. Arrows (▼, ► and ◄) indicate positions on the gels where the blots have been cropped. **(a)**, fifty μg of undigested lysates from ScN2a cells expressing 3F4-tagged WT and mutated MoPrPs was applied to each lane. Mut1 and Mut2 are ScN2a cell lysates expressing 3F4-tagged constructs pcDNA3.1::MoPrP(1-254, H61Y, H69Y, H77Y, H85Y) and pcDNA3.1::MoPrP(1-254, H61Y, H69Y, H77Y, H85Y, H96Y), respectively (not considered in this work). **(b)**, five hundred μg of cell lysate was digested with PK (20 μg/mL) at 37^o^C for 1 hour. H96Y* and H96Y** are ScN2a cell lysates expressing 3F4-H96Y mutant digested with 10 and 5 μg/mL PK, respectively. Mut1 and Mut2 constructs have not been considered in this work. **(c)** and **(e)**, full-length WB presented in Figure 4c. **(d)**, β-actin signals (arrows ► and ◄) on WB membrane previously probed with 3F4 antibody.


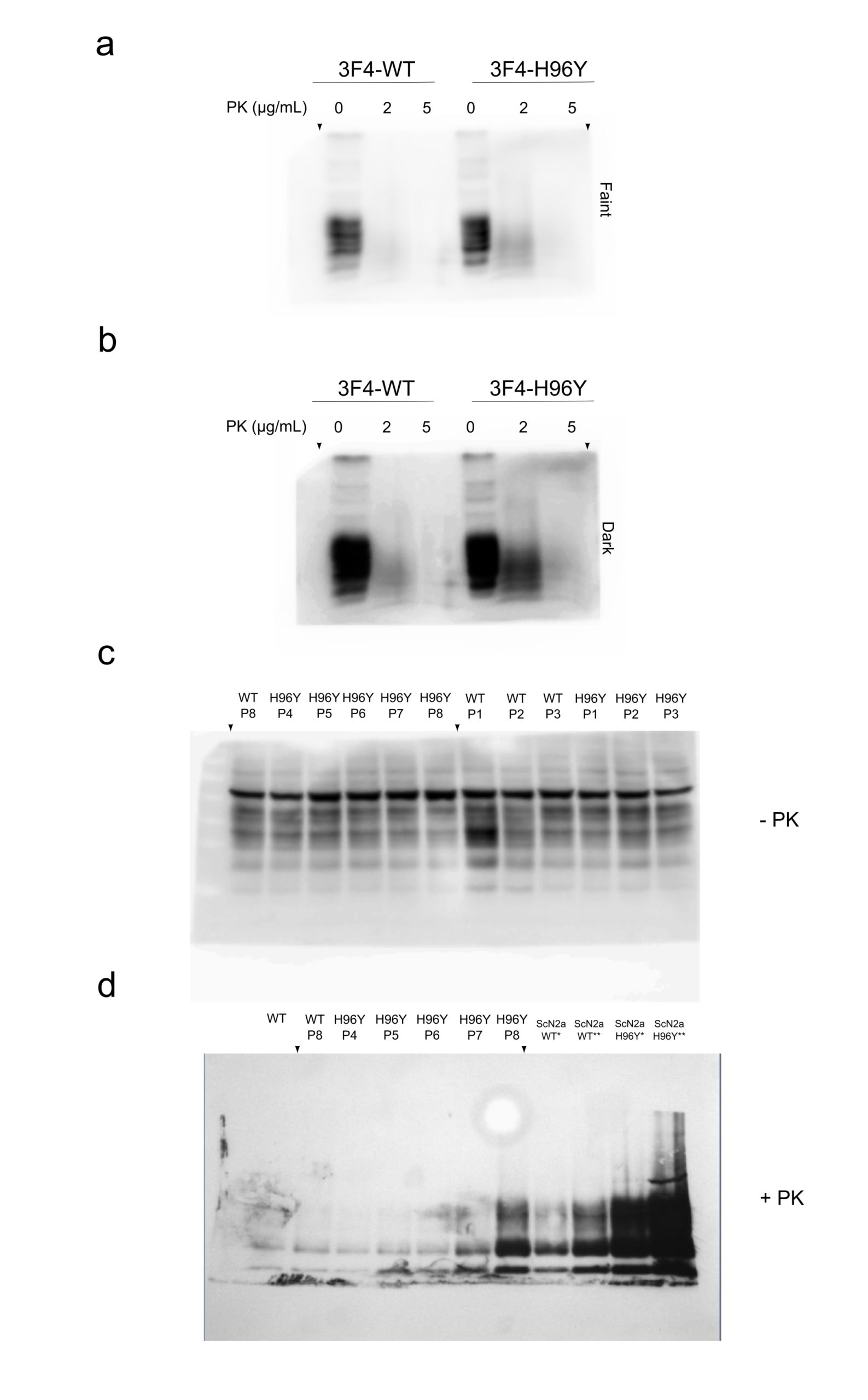


**Figure S5.** Full-length western-blots (WB) presented in Figure 5a and 5c in the main manuscript. Arrows (▼) indicate positions on the gels where the blots have been cropped. The H96Y mutant displays PK-resistance when expressed in N2a cells regularly passaged every 7 days up to passage (P) 8. Cell lysates were treated with 2 or 5 µg/mL of PK. Two exposures of the same blot are shown: **(a)** faint, 30 sec exposure; **(b)** dark, 6 min exposure. PTA-extracted PrP^Sc^ from N2a cells transfected with 3F4-H96Y MoPrP were inoculated into N2a cells and regularly passaged every 7 days up to P8. **(c)**, fifty μg of undigested lysates from N2a cells expressing 3F4-tagged WT and H96Y MoPrPs at different passages. **(d)**, the PrP^res^ detection was assessed by PK digestion (5 µg/mL) through passages (lines 1-7). ScN2a WT* and ScN2a WT** (lines 8-9) are ScN2a cell lysates expressing 3F4-WT MoPrP digested with 10 and 5 μg/mL PK, respectively. ScN2a H96Y* and ScN2a H96Y** (lines 10-11) are ScN2a cell lysates expressing 3F4-H96Y MoPrP digested with 10 and 5 μg/mL PK, respectively.

| Cu(II) WT pH=5.5 **(1)** | | | Cu(II) WT pH=7.0 **(2)** | | | Cu(II) Q212P pH=5.5 **(3)** | | | Cu(II) Q212P pH=7.0 **(4)** | | |
| --- | --- | --- | --- | --- | --- | --- | --- | --- | --- | --- | --- |
| **N** | **R (Å)** | **σ^2^ (Å^2^)** | **N** | **R (Å)** | **σ^2^ (Å^2^)** | **N** | **R (Å)** | **σ^2^ (Å^2^)** | **N** | **R (Å)** | **σ^2^ (Å^2^)** |
| 2 N_His_ | 1.98(2) | 0.006(3) | 1 N_His_ | 1.99(2) | 0.006(3) | 1 N_His_ | 2.00(2) | 0.007(3) | 1 N_His_ | 1.99(2) | 0.007(3) |
| 2 O/N | 1.98(2) | 0.008(3) | 3 O/N | 1.99(4) | 0.009(3) | 3 O/N | 1.99(2) | 0.009(3) | 3 O/N | 1.99(3) | 0.009(3) |
| 1 O | 2.31(3) | 0.013(4) | 1 O | 2.38(4) | 0.012(4) | 1 O | 2.40(4) | 0.013(4) | 1 O | 2.39(3) | 0.012(4) |
| 1 S | 3.25(4) | 0.013(4) | 1 S | 3.37(4) | 0.013(4) | 1 S | 3.47(4) | 0.014(4) | 1 S | 3.45(4) | 0.012(4) |
| Cu(II) P102L pH=5.5 **(5)** | | | Cu(II) P102L pH=7.0 **(6)** | | | Cu(II) H96Y pH=5.5 **(7)** | | | Cu(II) H96Y pH=7.0 **(8)** | | |
| **N** | **R (Å)** | **σ^2^ (Å^2^)** | **N** | **R (Å)** | **σ^2^ (Å^2^)** | **N** | **R (Å)** | **σ^2^ (Å^2^)** | **N** | **R (Å)** | **σ^2^ (Å^2^)** |
| 1 N_His_ | 2.00(3) | 0.006(3) | 1 N_His_ | 1.99(2) | 0.006(3) | 1 N_His_ | 2.00(2) | 0.007(3) | 1 N_His_ | 2.00(2) | 0.008(3) |
| O/N | 1.99(3) | 0.008(3) | 3 O/N | 1.99(3) | 0.009(3) | 3 O/N | 1.99(2) | 0.009(3) | 3 O/N | 1.99(2) | 0.008(3) |
| 1 O | 2.34(4) | 0.013(4) | 1 O | 2.38(3) | 0.012(4) | 1 O | 2.40(3) | 0.013(4) | 1 O | 2.39(3) | 0.012(4) |
| 1 S | 3.32(4) | 0.013(4) | 1 S | 3.38(4) | 0.013(4) | 1 S | 3.47(4) | 0.014(4) | 1 S | 3.45(4) | 0.013(4) |
| Cu(I) WT pH=5.5 **(9)** | | | Cu(I) WT pH=7.0 **(10)** | | | Cu(I) Q212P pH=5.5 **(11)** | | | Cu(I) Q212P pH=7.0 **(12)** | | |
| **N** | **R (Å)** | **σ^2^ (Å^2^)** | **N** | **R (Å)** | **σ^2^ (Å^2^)** | **N** | **R (Å)** | **σ^2^ (Å^2^)** | **N** | **R (Å)** | **σ^2^ (Å^2^)** |
| 2 N_His_ | 1.98(2) | 0.006(3) | 1 N_His_ | 1.98(2) | 0.007(3) | 1 N_His_ | 1.99(2) | 0.007(3) | 1 N_His_ | 1.99(2) | 0.007(3) |
| 2 O/N | 1.99(3) | 0.009(3) | 1 O/N | 2.00(2) | 0.009(3) | 1 O/N | 1.99(2) | 0.009(3) | 3 O/N | 1.99(3) | 0.009(3) |
| 1 O | 2.32(4) | 0.014(4) | 1 S | 2.27(4) | 0.009(4) | 1 S | 2.28(4) | 0.008(3) | 1 S | 2.27(3) | 0.009(4) |
| 1 S | 3.26(5) | 0.013(4) |  |  |  |  |  |  |  |  |  |
| Cu(I) P102L pH=5.5 **(13)** | | | Cu(I) P102L pH=7.0 **(14)** | | |  | | |  | | |
| **N** | **R (Å)** | **σ^2^ (Å^2^)** | **N** | **R (Å)** | **σ^2^ (Å^2^)** |  |  |  |  |  |  |
| 1 N_His_ | 2.00(2) | 0.009(3) | 1 N_His_ | 1.99(2) | 0.008(3) |  |  |  |  |  |  |
| 1 O/N | 1.99(2) | 0.009(3) | 3 O/N | 1.99(3) | 0.009(3) |  |  |  |  |  |  |
| 1 S | 2.28(4) | 0.009(3) | 1 O | 2.29(3) | 0.010(4) |  |  |  |  |  |  |

**Table S1. Structural parameters derived from the EXAFS analysis.** Structural parameters detemined from the fit of the EXAFS data at the Cu K-edge of samples 1 to 14. N is the coordination number, R is the distance between the copper ion and the ligand, σ^2^ is the Debye-Waller factor. Statistical errors are reported in parentheses. Codes 1 to 14 correspond to the following samples: Cu(II) WT HuPrP(90-231) pH5.5 **(1)**, Cu(II) WT HuPrP(90-231) pH 7.0 **(2)**, Cu(II) HuPrP(90-231, Q212P) pH 5.5 **(3)**, Cu(II) HuPrP(90-231, Q212P) pH 7.0 **(4)**, Cu(II) HuPrP(90-231, P102L) pH 5.5 **(5)**, Cu(II) HuPrP(90-231, P102L) pH 7.0 **(6)**, Cu(II) HuPrP(90-231, H96Y) pH 5.5 **(7)**, Cu(II) HuPrP(90-231, H96Y) pH 7.0 **(8)**, Cu(I) WT HuPrP(90-231) pH 5.5 **(9)**, Cu(I) WT HuPrP(90-231) pH 7.0 **(10)**, Cu(I) HuPrP(90-231, Q212P) pH 5.5 **(11)**, Cu(I) HuPrP(90-231, Q212P) pH 7.0 **(12)**, Cu(I) HuPrP(90-231, P102L) pH 5.5 **(13)**, Cu(I) HuPrP(90-231, P102L) pH 7.0 **(14)**
